# [RETRACTED ARTICLE]: Culture as a variable in neuroscience and clinical neuropsychology: A comprehensive review

**DOI:** 10.1590/1980-57642016dn11-030016

**Published:** 2017

José Roberto Wajman, Paulo Henrique Ferreira Bertolucci, Letícia Lessa Mansur, Serge Gauthier

The editorial board of the journal Dementia *&* Neuropsychologia communicates the formal retraction of the article:

Culture as a variable in neuroscience and clinical neuropsychology: a comprehensive review. *José Roberto Wajman, Paulo Henrique Ferreira Bertolucci, Letícia Lessa Mansur, Serge Gauthier.* Dement Neuropsychol 2015; 9(3):203-218.

This article is being retracted due to the identification of passages without due citation and others reproduced verbatim without quotation marks. ​The authors recognize these issues but the first author attributes them to erroneous submission of the wrong electronic version.

